# Proximal Lower Extremity Weakness Following Pedicle Subtraction Osteotomy in Adult Spinal Deformity: Influence of Correction Level and Clinical Outcomes

**DOI:** 10.7759/cureus.96160

**Published:** 2025-11-05

**Authors:** Shinsuke Sato, Yusuke Nakao, Shingo Kumaki, Shigeo Sano

**Affiliations:** 1 Orthopedic Surgery, Sanraku Hospital, Tokyo, JPN

**Keywords:** adult spinal deformity, pedicle subtraction osteotomy, proximal lower extremity muscle weakness, spinal deformity correction, spine surgery

## Abstract

Background

Neurological paralysis, particularly proximal lower extremity muscle weakness (PW) involving the iliopsoas and quadriceps, is a severe complication of surgery for adult spinal deformity (ASD) that significantly impairs ambulatory function and can delay postoperative rehabilitation and reduce quality of life. However, it remains unclear at which spinal level correction is most likely to induce PW. Therefore, this study aimed to identify the spinal level at which correction after ASD surgery is most likely to cause PW to help optimize surgical planning and postoperative recovery.

Methodology

To investigate the relationship between the pedicle subtraction osteotomy (PSO) correction level and the development of PW, we analyzed cases in which PSO was performed at a single vertebral level without changes to the alignment of other spinal segments. A total of 85 patients who underwent PSO for adult spinal deformity, including staged surgery or correction of iatrogenic kyphosis, were included. In staged cases, corrective procedures other than PSO were performed during the first stage without any weakness, and only PSO was performed during the second stage; patients who underwent PSO at the second stage were included in this analysis. Inclusion criteria were single-level PSO with complete pre- and postoperative radiographs and postoperative neurological assessment. Exclusion criteria were prior anterior/anterolateral spinal surgery and preoperative weakness of the iliopsoas or quadriceps. PW was defined as a manual muscle testing (MMT) score <3 for the iliopsoas and quadriceps muscles on postoperative day one. The incidence of PW was evaluated at each PSO level, and demographic characteristics and pre-/postoperative radiographic parameters were compared between the PW and non-PW groups.

Results

The vertebrae that underwent PSO were as follows: T8 (n = 1), T9 (n = 1), T11 (n = 1), T12 (n = 11), L1 (n = 6), L2 (n = 9), L3 (n = 15), L4 (n = 26), and L5 (n = 15). PW occurred in 20 patients (4 males, 16 females). PW occurred predominantly at the lower lumbar levels (L4-L5): 1/15 (6.7%) at L3, 12/26 (46.2%) at L4, and 7/15 (46.7%) at L5. PW resolved in all patients within one year of surgery, with the exception of one who was lost to follow-up. A significant difference was observed in the preoperative L4-S1 angle in the radiographic data (25.8° vs. 15.8°, p < 0.001).

Conclusions

PW occurred particularly frequently after the correction of kyphosis of the lower lumbar spine (L4 or L5), but the prognosis was favorable, with recovery in most cases within one year. Clinically, surgeons should anticipate this risk during lower lumbar correction and consider preventive strategies to reduce nerve root traction, such as minimizing prolonged hip extension intraoperatively and maintaining hip and knee flexion postoperatively in the early postoperative period.

## Introduction

Neurological paralysis, particularly proximal lower extremity muscle weakness (PW) involving the iliopsoas and quadriceps, is a severe complication of adult spinal deformity (ASD) surgery that can significantly affect ambulatory function [1-3]. Reported incidences of neurological deficits after ASD surgery range from 5% to 20%, depending on deformity severity and surgical invasiveness [1,2,4,5]. Such deficits are often attributed to nerve root traction, ischemia, or mechanical compression during deformity correction [4]. These complications may prolong rehabilitation and negatively influence long-term outcomes, underscoring their clinical importance [1-3,5].

While several studies have discussed global correction parameters associated with postoperative weakness, few have analyzed which specific spinal level correction most strongly predisposes patients to proximal weakness [6]. In particular, pedicle subtraction osteotomy (PSO) can create substantial angular correction at a single level, leading to significant lengthening of anterior column structures and potential stretching of the lumbar plexus [7,8]. Identifying the level most associated with PW could guide surgical planning and inform postoperative management to mitigate risk.

Furthermore, intraoperative monitoring with motor evoked potentials (MEPs) is widely used to reduce the risk of neurological complications [9,10]. However, postoperative weakness can still occur despite stable intraoperative MEPs, suggesting mechanisms beyond intraoperative ischemic injury, such as delayed traction neuropathy [11-13]. Similar delayed-onset nerve dysfunctions have been reported in the context of cervical decompression-related C5 palsy and lumbar nerve root palsy after osteotomy [13,14], supporting the possibility that postoperative deficits may arise from traction-related mechanisms rather than direct intraoperative injury.

Therefore, this study aimed to clarify which spinal level correction during ASD surgery is most likely to induce proximal lower extremity weakness and investigate the impact of postoperative hip flexion maintenance (using a pillow) in preventing PW after surgery.

## Materials and methods

This retrospective study included 85 patients who underwent PSO at a single vertebral level between 2018 and 2024. Cases with other spinal alignment changes or previous anterior spinal surgery were excluded.

Among the included cases, intraoperative MEPs did not decrease by more than 70%. Only cases in which PSO was the sole procedure at the analyzed stage were included to isolate the effect of PSO level on postoperative weakness. The cohort, therefore, included staged surgeries in which corrective procedures other than PSO were performed during the first stage without any weakness, and only PSO was performed during the second stage; patients who underwent PSO at the second stage were included in this analysis.

PW was defined as a manual muscle testing (MMT) score of less than 3 for the iliopsoas or quadriceps on postoperative day one. The evaluation was performed on the first postoperative day rather than immediately after surgery because the degree of awakening from anesthesia varies among patients, making immediate postoperative assessment less reliable and less consistent. As no standardized definition for PW after ASD surgery exists, this threshold represents our institution’s a priori operational definition to capture clinically meaningful weakness.

**Figure 1:**
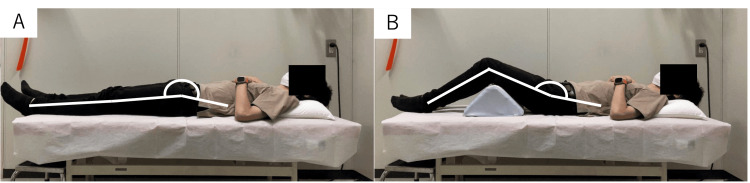
Correct use of the pillow. (A) Lateral view of the supine position. The knee is fully extended, and the hip is extended. (B) The hip joint is flexed by placing a pillow under the knee.

To prevent excessive nerve root tension after correction, a pillow was placed under the knees for approximately one week postoperatively, keeping the hips and knees flexed to reduce mechanical traction on the lumbar plexus (L1-4 roots) (Figure 1). This correction pillow protocol was institutionally implemented in March 2023; thereafter, all consecutive patients routinely received the positioning, whereas patients operated before March 2023 did not. These time-defined cohorts constituted the “after” (≥March 2023) and “before” (<March 2023) groups, respectively. The incidence of PW was compared between the before/after cohorts and between the PW and non-PW groups.

The following parameters were compared between the PW and non-PW groups: age, body mass index (BMI), operative time, estimated blood loss (EBL), correction angle achieved by PSO, pelvic incidence (PI), preoperative and postoperative pelvic tilt (PT), L1-S1 lordosis (LL), L4-S1 lordosis (LLL), thoracic kyphosis (TK), and T1 pelvic angle (TPA).

Statistical analysis

Continuous variables were expressed as medians with interquartile ranges (IQRs) and compared using the Mann-Whitney U test. Categorical variables were analyzed using Fisher’s exact test or the chi-square test as appropriate. A p-value <0.05 was considered statistically significant. Statistical analyses were performed using R (version 4.3.0; The R Foundation for Statistical Computing, Vienna, Austria).

## Results

The vertebrae that underwent PSO were as follows: T8 (n = 1), T9 (n = 1), T11 (n = 1), T12 (n = 11), L1 (n = 6), L2 (n = 9), L3 (n = 15), L4 (n = 26), and L5 (n = 15). PW occurred in 20 patients (4 males, 16 females). Patient characteristics and surgical details of the PW and non-PW groups are presented in Table 1. No cases of PW were observed in patients who underwent PSO at the thoracic spine or at the L1 or L2 levels. One (6.7%), 12 (46.2%), and 7 (46.7%) patients experienced PW at L3, L4, and L5, respectively. Overall, PW occurred predominantly at the lower lumbar levels (L4-L5). Because several levels had zero events, across-level hypothesis testing was restricted to L3-L5; this comparison demonstrated a significant difference in incidence across levels (chi-square test, p = 0.023). Interestingly, delayed PW onset (occurring on postoperative days 2-7) was observed in one, three, and one patient after PSO at L3, L4, and L5, respectively.

**Table 1:** Comparison of PW and non-PW groups. The values are presented as mean ± SD. *: p < 0.05. **: p < 0.005. BMI = body mass index; EBL = estimated blood loss; LL = L1-S1 lordosis; LLL = L4-S1 lordosis; PI = pelvic index; PT = pelvic tilt; PW = proximal lower extremity muscle weakness; TK = thoracic kyphosis; TPA = T1 pelvic angle

	Non-PW	PW	PW vs. non-PW p-value
Age	75.8 ± 0.7	72.3 ± 1.0	0.0884
BMI	25.0 ± 4.9	24.6 ± 4.2	0.875
Operation time	187.1 ± 54.4	169.3 ± 36.7	0.252
EBL	839.5 ± 432.8	787.5 ± 356.2	0.809
PI	49.8 ± 12.3	48.8 ± 9.6	0.709
Preoperative PT	29.5 ± 11.2	32.3 ± 5.5	0.0868
Preoperative LL	29.1 ± 16.6	25.6 ± 12.6	0.431
Preoperative LLL	25.8 ± 12.9	15.8 ± 7.8	0.000882**
Preoperative TK	47.2 ± 16.3	43.9 ± 15.3	0.306
Preoperative TPA	28.4 ± 11.2	29.4 ± 7.3	0.369
Preoperative PI-LL	20.7 ± 10.3	23.3 ± 11.0	0.64
Correction angle	22.5 ± 8.4	22.2 ± 5.1	0.866
Postoperative PT	21.0 ± 8.2	19.4 ± 6.2	0.32
Postoperative LL	21.0 ± 8.2	46.2 ± 11.0	0.789
Postoperative LLL	46.7 ± 12.6	37.2 ± 7.7	0.189
Postoperative TK	49.1 ± 15.4	47 ± 14.3	0.663
Postoperative TPA	16.8 ± 6.9	15.9 ± 5.1	0.577
Postoperative PI-LL	3.1 ± 12.8	2.6 ± 10.3	0.686

The details of the 20 patients with PW are shown in the Appendices. Despite the relatively high incidence at L4-L5, the deficit was mostly transient, with complete recovery within one year in all patients followed. Two cases of tibialis anterior weakness occurred during PSO at L5. One patient had left-sided weakness (MMT score = 1), which resolved completely within six months. The other developed bilateral weakness (MMT score = 2), which persisted for two years. All patients experienced varying degrees of sensory disturbances, including anterior thigh pain or tingling sensations. No structural compression was identified on postoperative CT or MRI, supporting a traction rather than compression mechanism.

Among the preoperative and postoperative factors, only preoperative L4-S1 lordosis differed significantly between groups (25.8° vs 15.8°, p < 0.001) (Table 1). For level-specific analyses, at L4 PSO, the PW group was older than the non-PW group (69.2 ± 8.5 vs. 75.5 ± 3.2, p = 0.0192) (Table 2). In contrast, at L5 PSO, there were no significant between-group differences in age or other parameters (all p ≥ 0.0726) (Table 3). These findings suggest that older age may predispose to PW after L4 PSO, whereas no such association was evident at L5.

**Table 2:** Comparison of PW and non-PW groups at L4 PSO. The values are presented as mean ± SD. *: p < 0.05 is considered significant BMI = body mass index; EBL = estimated blood loss; LL = L1-S1 lordosis; LLL = L4-S1 lordosis; PI = pelvic index; PSO = pedicle subtraction osteotomy; PT = pelvic tilt; PW = proximal lower extremity muscle weakness; TK = thoracic kyphosis; TPA = T1 pelvic angle

	PW (L4)	Non-PW (L4)	PW (L4) vs. non-PW (L4) p-value
Age	69.2 ± 8.5	75.5 ± 3.2	0.0192*
BMI	26.6 ± 6.2	24.9 ± 3.3	0.86
Operation time	199.8 ± 61.6	170.8 ± 31.0	0.354
EBL	772.1 ± 251.6	863.8 ± 383.3	0.781
PI	46.9 ± 14.1	46.8 ± 8.8	0.959
Preoperative PT	28.1 ± 9.2	33.4 ± 4.3	0.0371
Preoperative LL	23.9 ± 18.2	25.4 ± 9.0	0.918
Preoperative LLL	15.8 ± 10.1	15.4 ± 7.3	0.877
Preoperative TK	38.1 ± 11.3	41.7 ± 11.2	0.455
Preoperative TPA	29.4 ± 8.6	27.9 ± 6.2	0.877
Preoperative PI-LL	20.9 ± 11.5	21.3 ± 8.6	0.68
Correction angle	21.6 ± 6.3	22.7 ± 4.9	0.571
Postoperative PT	18.3 ± 6.9	18.6 ± 5.5	0.979
Postoperative LL	45.3 ± 12.7	46.7 ± 7.1	0.918
Postoperative LLL	36.4 ± 7.2	38.6 ± 8.8	0.757
Postoperative TK	42.5 ± 11.9	46.0 ± 10.9	0.487
Postoperative TPA	15.4 ± 5.8	15.3 ± 4.6	0.979
Postoperative PI-LL	1.6 ± 7.7	0.1 ± 6.6	0.624

**Table 3:** Comparison of PW and non-PW groups at L5 PSO. The values are presented as mean ± SD. *: p < 0.05 is considered significant BMI = body mass index; EBL = estimated blood loss; LL = L1-S1 lordosis; LLL = L4-S1 lordosis; PI = pelvic index; PSO = pedicle subtraction osteotomy; PT = pelvic tilt; PW = proximal lower extremity muscle weakness; TK = thoracic kyphosis; TPA = T1 pelvic angle

	PW (L5)	Non-PW (L5)	PW (L5) vs. non-PW (L5) p-value
Age	66.4 ± 10.4	75.4 ± 2.8	0.147
BMI	23.4 ± 3.7	24.3 ± 5.4	0.867
Operation time	161.0 ± 20.6	168.6 ± 45.1	0.602
EBL	658.8 ± 227.1	640.7 ± 237.8	0.779
PI	51.0 ± 16.9	51.3 ± 10.0	0.772
Preoperative PT	38.8 ± 11.6	31.7 ± 6.3	0.523
Preoperative LL	20.8 ± 8.3	23.6 ± 16.4	0.487
Preoperative LLL	9.9 ± 7.3	14.3 ± 6.6	0.324
Preoperative TK	37.8 ± 13.4	46.6 ± 20.3	0.336
Preoperative TPA	36.0 ± 13.7	32.3 ± 8.2	0.685
Preoperative PI-LL	12.3 ± 9.5	27.7 ± 13.3	0.0726
Correction angle	26.4 ± 9.1	21.9 ± 5.1	0.245
Postoperative PT	26.5 ± 8.9	20.6 ± 7.2	0.293
Postoperative LL	42.6 ± 16.2	43.6 ± 14.8	0.779
Postoperative LLL	35.8 ± 5.3	35.7 ± 3.9	0.683
Postoperative TK	42.1 ± 10.8	47.0 ± 18.5	0.45
Postoperative TPA	19.4 ± 9.1	16.1 ± 5.5	0.449
Postoperative PI-LL	8.4 ± 15.7	7.7 ± 13.5	0.728

A comparison of the incidence of muscle weakness before and after the introduction of the corrective pillow is presented in Table 4.

**Table 4:** Comparison of PW incidence before and after the introduction of the correction pillow. The values are presented as % (N). PSO = pedicle subtraction osteotomy; PW = proximal lower extremity muscle weakness

PSO level	Before	After
Thoracic	0% (0/13)	0% (0/1)
L1	0% (0/6)	-
L2	0% (0/7)	0% (0/2)
L3	7.1% (1/7)	0% (0/1)
L4	47.6% (10/21)	40% (2/5)
L5	57.1% (4/7)	37.5% (3/8)

Representative case

**Figure 2:**
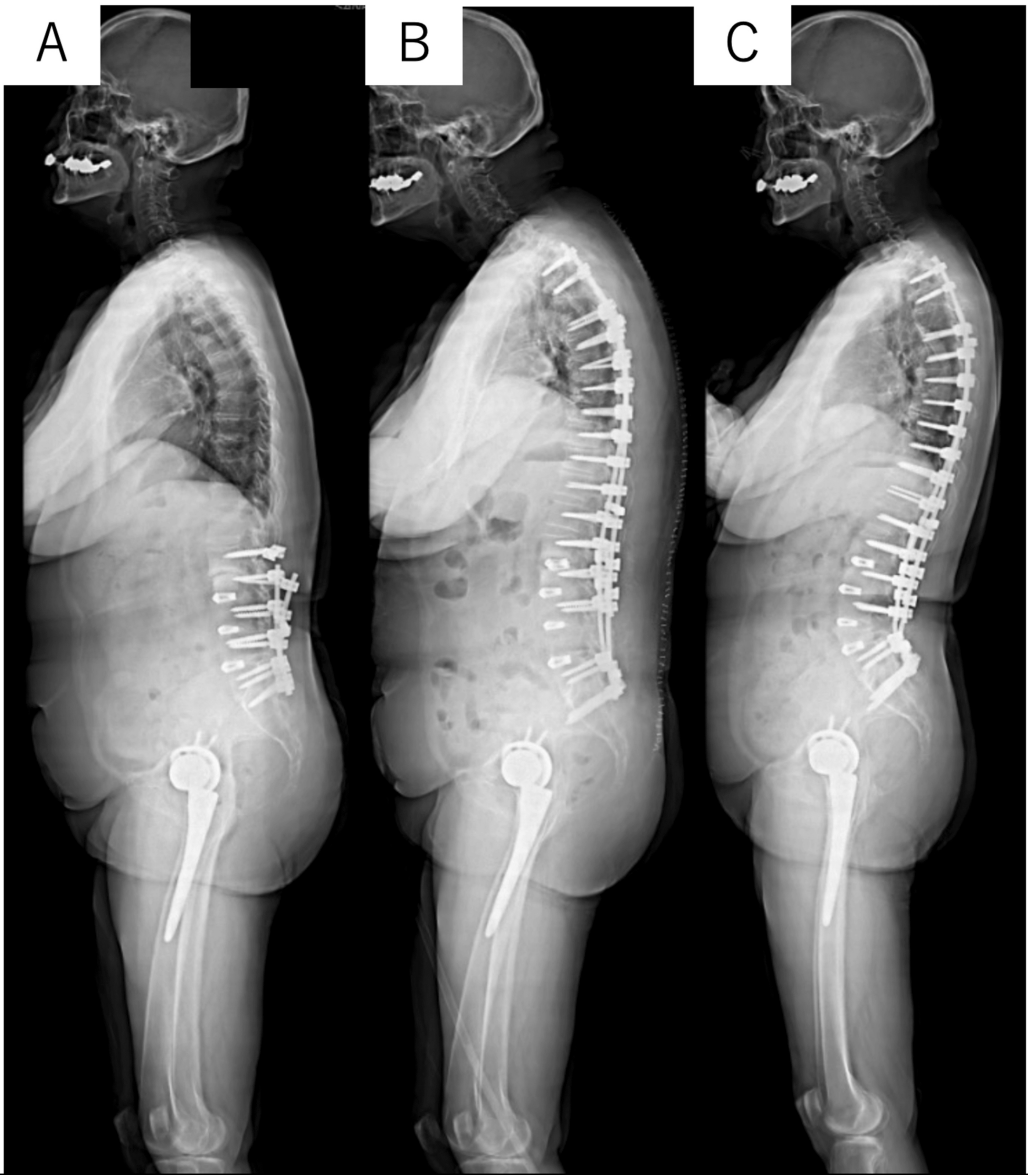
Standing lateral radiograph of the entire spine of a 72-year-old woman. (A) Preoperative standing lateral radiograph of the entire spine. Iatrogenic kyphosis, a fixed lumbar spine, and minimal kyphosis are present. (B) Postoperative (posterior corrective fixation of T2–S2) standing lateral radiograph of the entire spine. (C) Postoperative (pedicle subtraction osteotomy at L5) standing lateral radiograph of the entire spine. Good spinal alignment is observed.

A 75-year-old woman presented with iatrogenic spinal deformity, including mild kyphosis in the lower lumbar region, after undergoing multiple surgeries at another hospital (Figure 2). As a result, she underwent two-stage corrective surgery. In the first stage, posterior corrective fusion from T2 to the pelvis was performed, with no postoperative neurological complications. Two weeks later, a PSO was performed at L5 with a local correction angle of 26°. This resulted in postoperative weakness of the iliopsoas and quadriceps muscles (MMT score = 2). However, the weakness gradually improved, and full muscle strength (MMT score = 5) was regained within four months.

## Discussion

The causes of nerve palsy during deformity surgery include ischemia, compression, and traction [4]. C5 palsy is typically attributed to posterior traction of the nerve root [6,7], whereas sciatic or femoral nerve palsy after total hip arthroplasty is often caused by leg lengthening [8]. Nerve traction has, therefore, been considered a key mechanism of iatrogenic nerve injury. Consistent with these mechanisms, our results, showing a higher incidence of PW at L4-L5 and no compressive lesions on postoperative imaging, suggest that traction likely plays a dominant role in postoperative weakness after lower lumbar PSO. In the present study, across 85 cases, no intraoperative MEP decrease >70% was observed, making direct intraoperative neural manipulation less likely to be the primary cause of PW. Instead, PW after PSO may result from muscle weakness secondary to nerve root traction during corrective procedures [12,14].

**Figure 3:**
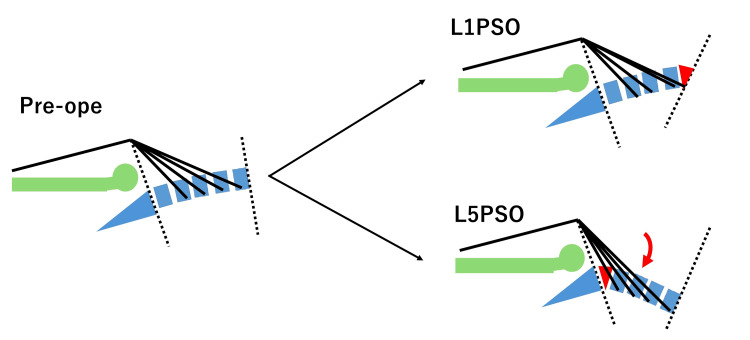
Supine lateral schema showing the mechanism of nerve root lengthening. Pedicle subtraction osteotomy (PSO) at L1 does not lengthen the L1–L4 root. PSO at L1 does not significantly change the length of the L1–L4 nerve roots. In contrast, PSO at L5 results in increased hip extension and posterior stretching of these nerve roots due to the correction. Image credits: Shinsuke Sato.

Corrective surgery increases lumbar lordosis and pelvic anteversion, producing relative hip extension that may stretch the L1-4 roots, particularly the femoral (L2-4) component [11,13,15-17]. Cadaveric measurements have demonstrated measurable elongation of the L4 root during the femoral nerve stretch test, supporting the plausibility of traction with hip extension after sagittal realignment [11]. Figure 3 schematically illustrates how lower lumbar correction (e.g., L5 PSO) increases root angulation/length compared with L1 correction, which may contribute to iliopsoas and quadriceps weakness.

Because patients remain in the supine posture for a prolonged period immediately after surgery, the influence of corrective surgery on nerve root tension was considered based on this position. For example, PSO at L1 does not substantially change the length of the L1-4 roots compared with preoperative measurements, whereas PSO at L5 elongates the roots due to relative hip extension and posterior angulation (Figure 3). Thus, lower lumbar PSO (such as at L4 and L5) may stretch the nerve roots to a greater extent, explaining the higher incidence of PW at these levels.

This phenomenon is not limited to PSO; it may also explain PW during correction of lumbar kyphosis in procedures without osteotomy, particularly when correcting lower lumbar deformities. PW was predominantly observed in patients with slight preoperative L4-S1 lordosis (i.e., those who required PSO at L4 or L5), and a large proportion of PW cases involved the L4 and L5 levels. When PW and non-PW groups were compared, a significant age difference was observed among patients who underwent PSO at L4, whereas no significant differences were found for other characteristics. Therefore, PSO at L4 or L5 may lead to PW, largely independent of baseline characteristics. These results underscore the need for preventive measures, such as early postoperative hip/knee flexion, especially when correcting lower lumbar deformities.

The prognosis of PW was favorable: muscle strength generally recovered within 12 months (median time to full recovery: six months); in our cohort, all patients recovered by one year, except one lost to follow-up. This pattern is consistent with previous reports of lumbar nerve root palsy and postoperative femoral neuropathy after spine surgery [4], as well as recovery patterns reported after nerve palsy following total hip arthroplasty [8].

Delayed onset of PW in some patients suggests that the duration of root extension may contribute to postoperative weakness, similar to delayed C5 palsy after cervical spine surgery [6,7]. Intraoperative MEPs may not have detected these subtle traction injuries because the duration of root elongation was insufficient during surgery. To mitigate this risk, a correction pillow was placed under the knees for approximately one week postoperatively, maintaining hip and knee flexion to reduce time spent in relative hyperextension. Following the institutional implementation of this protocol in March 2023, the incidence of PW showed a numerical decrease at the lower lumbar levels, with a fall from 47.6% to 40% at L4 and from 57.1% to 37.5% at L5. However, the study was not powered to detect small differences between periods. Notably, no delayed PW was observed after adoption of the correction pillow protocol. These findings highlight the importance of intraoperative positioning and early postoperative management in preventing traction-related nerve injuries and warrant prospective validation of the pillow intervention.

Several limitations should be acknowledged. First, the lack of quantitative assessment of nerve root lengthening represents a major limitation, as hip joint angles vary substantially with posture (e.g., sitting vs. standing), making precise measurement difficult. Second, although no radiological compression or MEP decrease greater than 70% was observed, we cannot completely exclude the possibility that intraoperative manipulation contributed to PW [18]. Third, the sample size per level, particularly at T8-L2, was small, which limits the precision of level-specific estimates and reduces generalizability. Finally, the single-institution, retrospective design introduces potential selection bias and may limit external validity. Further studies incorporating quantitative intraoperative nerve monitoring or imaging, larger multicenter cohorts, and prospective validation of preventive strategies may help clarify the mechanisms underlying PW and refine management.

## Conclusions

PW occurred in approximately half of the patients with PSO at L4 or L5; however, the prognosis for patients with weakness was good, probably because of the L1-4 root lengthening by PSO correction. PW can occur not only during PSO but also during deformity surgery without osteotomy, particularly when kyphosis of the lower lumbar spine is present.

## Appendices

**Table 5:** Clinical characteristics of the patients in the PW group. Bi = bilateral; IP = iliopsoas; L = left; R = right; MMT = manual muscle testing; PO = postoperative; PSO = pedicle subtraction osteotomy; PW = proximal lower extremity muscle weakness; Quad = quadriceps

PSO level	PW side	MMT of IP	MMT of Quad	Delayed onset	prognosis
L3	R	2-	1+	PO 2 days	1 year MMT2 (lost to follow-up)
L4	Bi	2	2	-	Full recovery at PO 1 month
L4	L	-	2-	-	Full recovery at PO 1 year
L4	R	2	-	-	Full recovery at PO 6 months
L4	L	1+	1+	-	Full recovery at PO 1 year
L4	Bi	2	-	-	Full recovery at PO 6 months
L4	Bi	2	2	-	Full recovery at PO 6 months
L4	Bi	2-	2-	-	Full recovery at PO 1 year
L4	Bi	2	2	-	Full recovery at PO 3 months
L4	L	2-	-	-	Full recovery at PO 2 months
L4	L	2	2	PO 5 days	Full recovery at PO 3 months
L4	R	-	2	PO 3 days	Full recovery at PO 8 months
L4	R	2	2	PO 4 days	Full recovery at PO 5 months
L5	L	2	-	-	Full recovery at PO 7 months
L5	R	2	-	-	Full recovery at PO 8 months
L5	R	2	-	-	Full recovery at PO 6 months
L5	Bi	2-	-	-	Full recovery at PO 4 months
L5	Bi	2	-	-	Full recovery at PO 6 months
L5	R	2	-	-	Full recovery at PO 2 months
L5	R	2-	-	PO 7 days	Full recovery at PO 1 month
